# Supplementary material for: High-Intensity Focused Ultrasound Decreases Subcutaneous Fat Tissue Thickness by Increasing Apoptosis and Autophagy
Source: Biomolecules. 2023 Feb 18;13(2):392. doi: 10.3390/biom13020392 (PMC9953651; doi:10.3390/biom13020392)
Supplement: Supplementary file 1 [file biomolecules-13-00392-s001.zip › biomolecules-2225705-supplementary.pdf]

biomolecules

MDPI

Article

High-Intensity Focused Ultrasound Decreases Subcutaneous Fat Tissue Thickness by Increasing Apoptosis and Autophagy

Kyung-A Byun [1,2,†], Hyun Jun Park [3,†], Seyeon Oh [2], Sosorburam Batsukh [1,2], Hye Jin Sun [4], Taehui Kim [4], Sunggeun Kim [4], Donghwan Kang [4], Kuk Hui Son [5,*] and Kyunghee Byun [1,2,*]

1 Department of Anatomy & Cell Biology, College of Medicine, Gachon University, Incheon 21936, Republic of Korea
2 Functional Cellular Networks Laboratory, Lee Gil Ya Cancer and Diabetes Institute, Gachon University of Medicine, Incheon 21999, Republic of Korea
3 Maylin Anti-Aging Center Apgujeong, Seoul 06005, Republic of Korea
4 Jeisys Medical Inc., Seoul 08501, Republic of Korea
5 Department of Thoracic and Cardiovascular Surgery, Gachon University Gil Medical Center, Gachon University, Incheon 21565, Republic of Korea
† These authors contributed equally to this work as co-first authors of this study.
* Correspondence: dr632@gilhospital.com (K.H.S.); khbyun1@gachon.ac.kr (K.B.); Tel.: +82-32-460-3666 (K.H.S.); +82-32-899-6511 (K.B.)

Table S1. List of antibodies used for western blot.

Antigen (host)	Company	Catalog no.	Dilution rate
p53 (Moues)	Santa cruz biotechnology	Sc-126	1:1,000
Histon H1 (Rabbit)	Abclonal	A4342	1:1,000
COX IV (Mouse)	Cell signaling technology	11967	1:1,000
β-actin (Rabbit)	Cell signaling technology	4967	1:1,000
BCL2 (Rabbit)	Abclonal	A11313	1:1,000
BCL-xL (Moues)	Santa cruz biotechnology	Sc-8392	1:500
BAX (Rabbit)	Cell signaling technology	2774	1:1,000
BAK (Rabbit)	Abclonal	A0498	1:1,000
Cyto C (Rabbit)	Cell signaling technology	4280	1:1,000
Caspase 3 (Rabbit)	Cell signaling technology	9662	1:1,000
Caspase 9 (Mouse)	Cell signaling technology	9508	1:1,000
ATG5 (Rabbit)	Novus Biological	NB110-53818	1:500
BECN1 (Mouse)	Santa cruz biotechnology	Sc-48341	1:1,000
P62 (Rabbit)	Gene Tex	GTX102359	1:1,000
LC3 (Rabbit)	Cell signaling technology	4108	1:1,000

Table S2. List of primer for quantitative real time polymerase chain reaction. (qRT-PCR).

Gene		Primers
Actb	Forward	5'-CCG TAA AGA CCT CTA TGC CAA C-3′
	Reverse	5'-GCA GTA ATC TCC TTC TGC ATC C-3′
Notch1	Forward	5'-TAA TTG CCA GAC CAA CAT CAA C-3′
	Reverse	5'-CAC TTG TAT CCA GCG ACA TCA T-3′

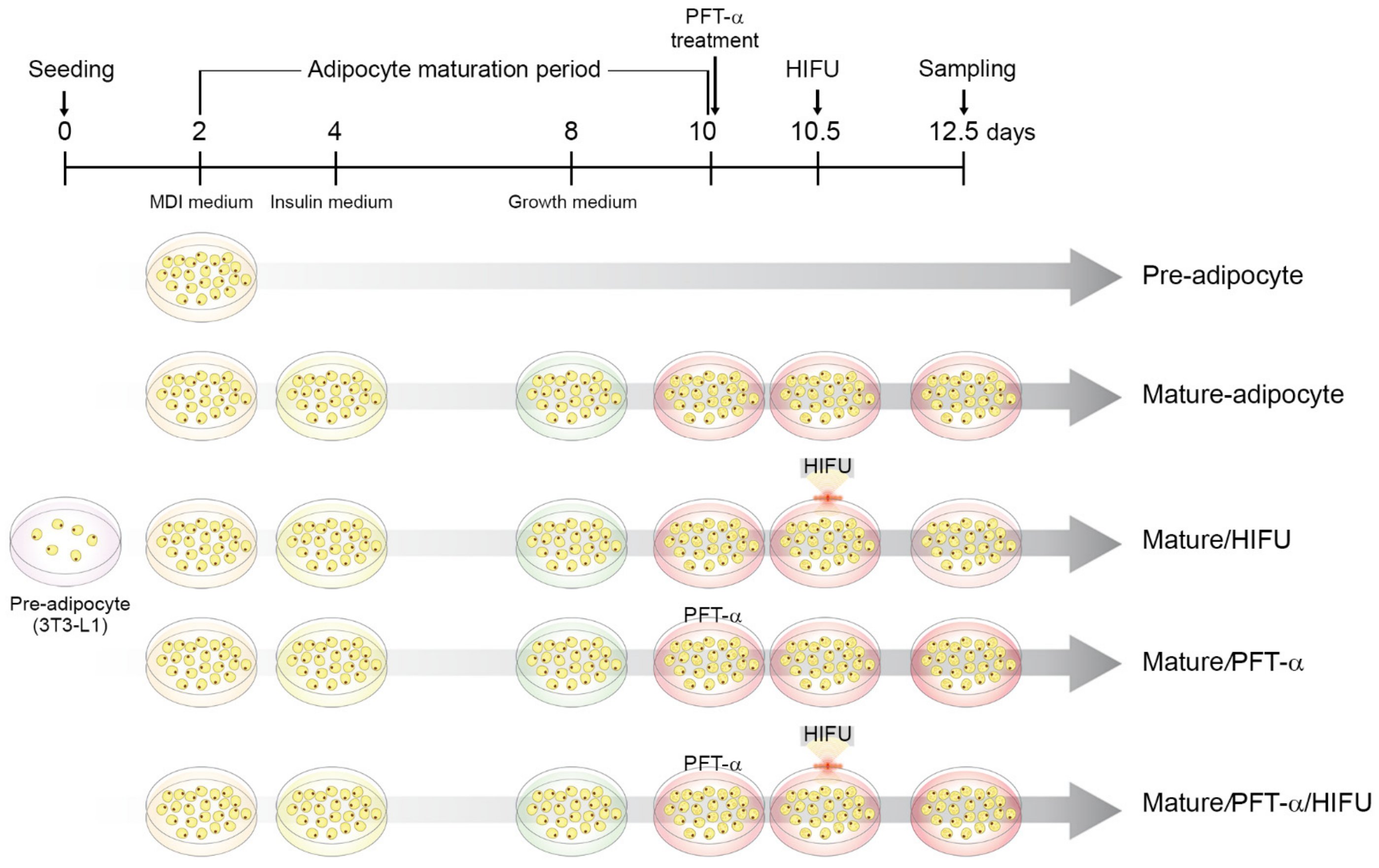

Figure S1. In vitro model schematic summary.

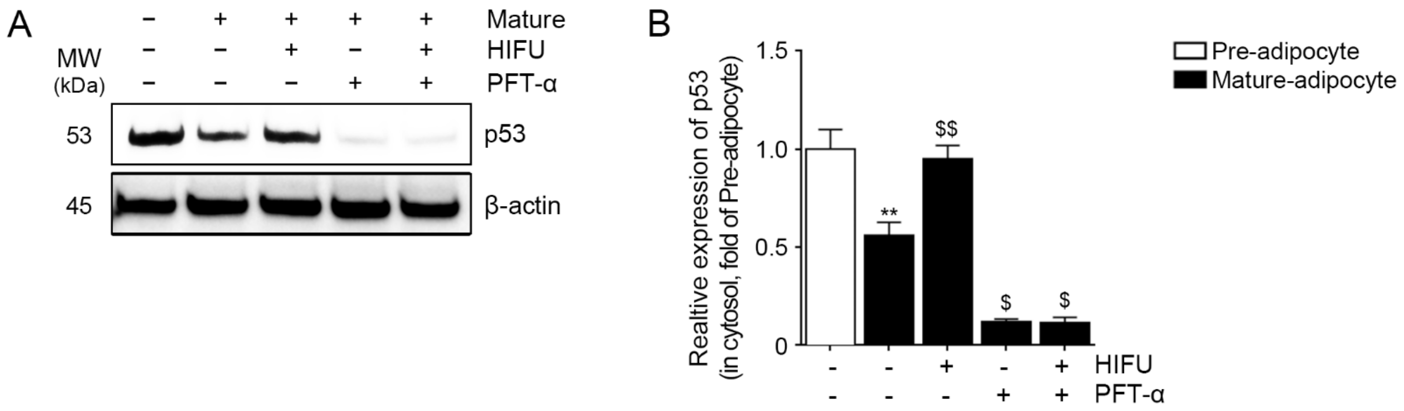

Figure S2. Regulation of the p53 in adipocytes by HIFU treatment. (A) The protein expression levels of p53 were analyzed by western blot. (B) The quantitative graphs represent the results of the western blot analysis. The protein expression level of p53 was increased by HIFU treatment and decreased modulating p53. The data are presented as the mean ± standard deviation; **, $p < 0.01$ Mature-adipocyte *vs*. Pre-adipocyte; $, $p < 0.05$ or $$, $p < 0.01$ *vs*. Mature-adipocyte (Mann–Whitney U test). HIFU, high-intensity focused ultrasound; MW, molecular weight; PFT-*α*, pifithrin-*α* hydrobromide.

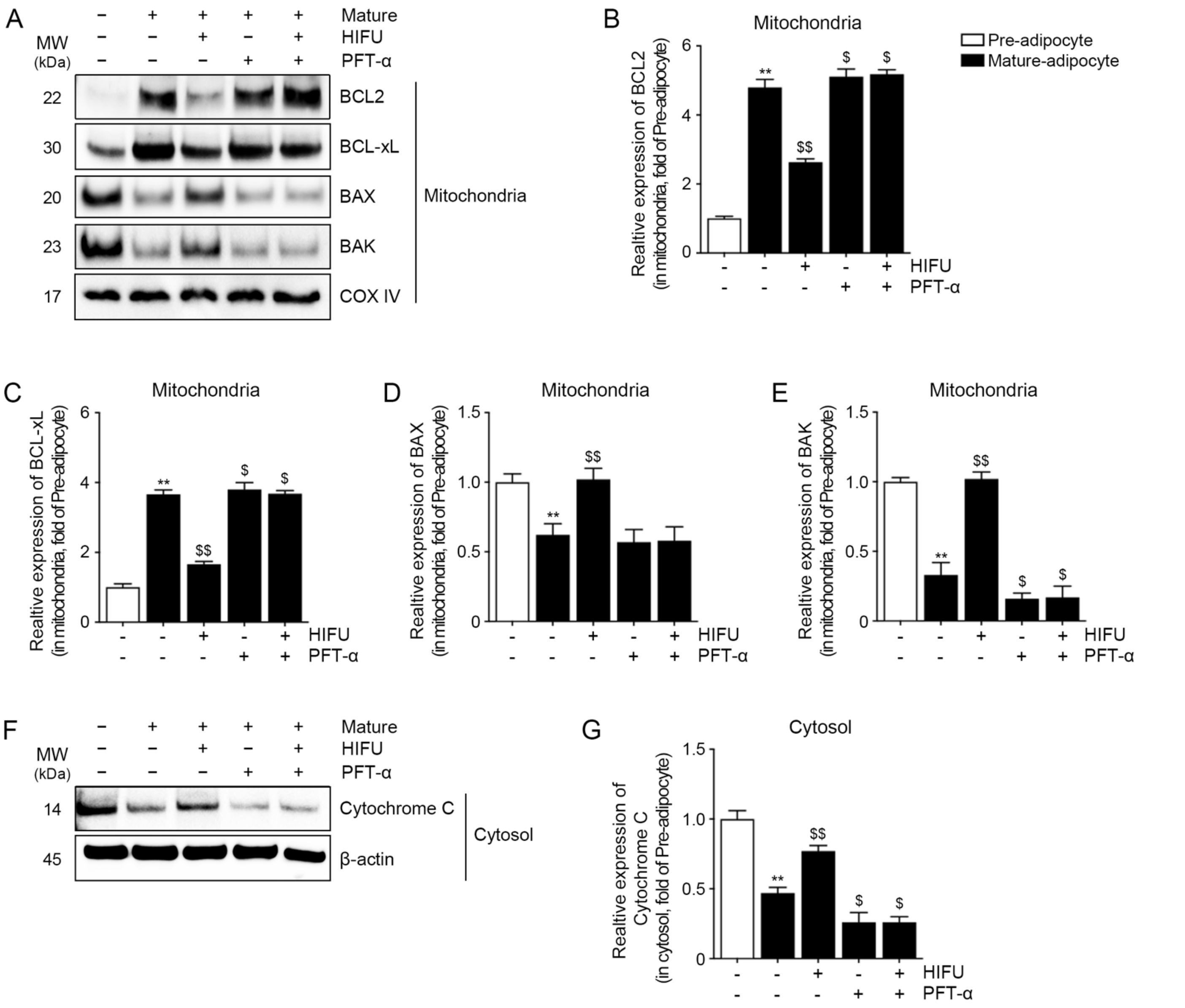

Figure S3. Regulation of the Bcl-2 family and cytochrome C in adipocytes by HIFU treatment. (A) The protein expression levels of Bcl-2 family members were analyzed by western blot. (B–E) The quantitative graphs represent the results of the western blot analysis. The protein expression levels of BCL2 (B) and BCL-xL (C) were decreased by HIFU treatment and increased modulating p53. The protein expression levels of BAX (D) and BAK (E) were increased by HIFU treatment and decreased modulating p53. (F) The protein expression of Cyto C was analyzed by western blot. (G) The quantitative graphs represent the results of the western blot analysis. The protein expression of Cyto C was increased by HIFU treatment and decreased modulating p53. The data are presented as the mean ± standard deviation; **, $p < 0.01$ Mature-adipocyte *vs.* Pre-adipocyte; $, $p < 0.05$ or $$, $p < 0.01$ *vs.* Mature-adipocyte (Mann–Whitney U test). BAK, Bcl-2 homologous antagonist/killer; BAX, bcl-2-like protein 4; BCL2, B-cell lymphoma 2; BCL-xL, B-cell lymphoma-extra large; COX IV, cytochrome c oxidase subunit 4; Cyto C, cytochrome C; HIFU, high-intensity focused ultrasound; MW, molecular weight; PFT-α, pifithrin-α hydrobromide.

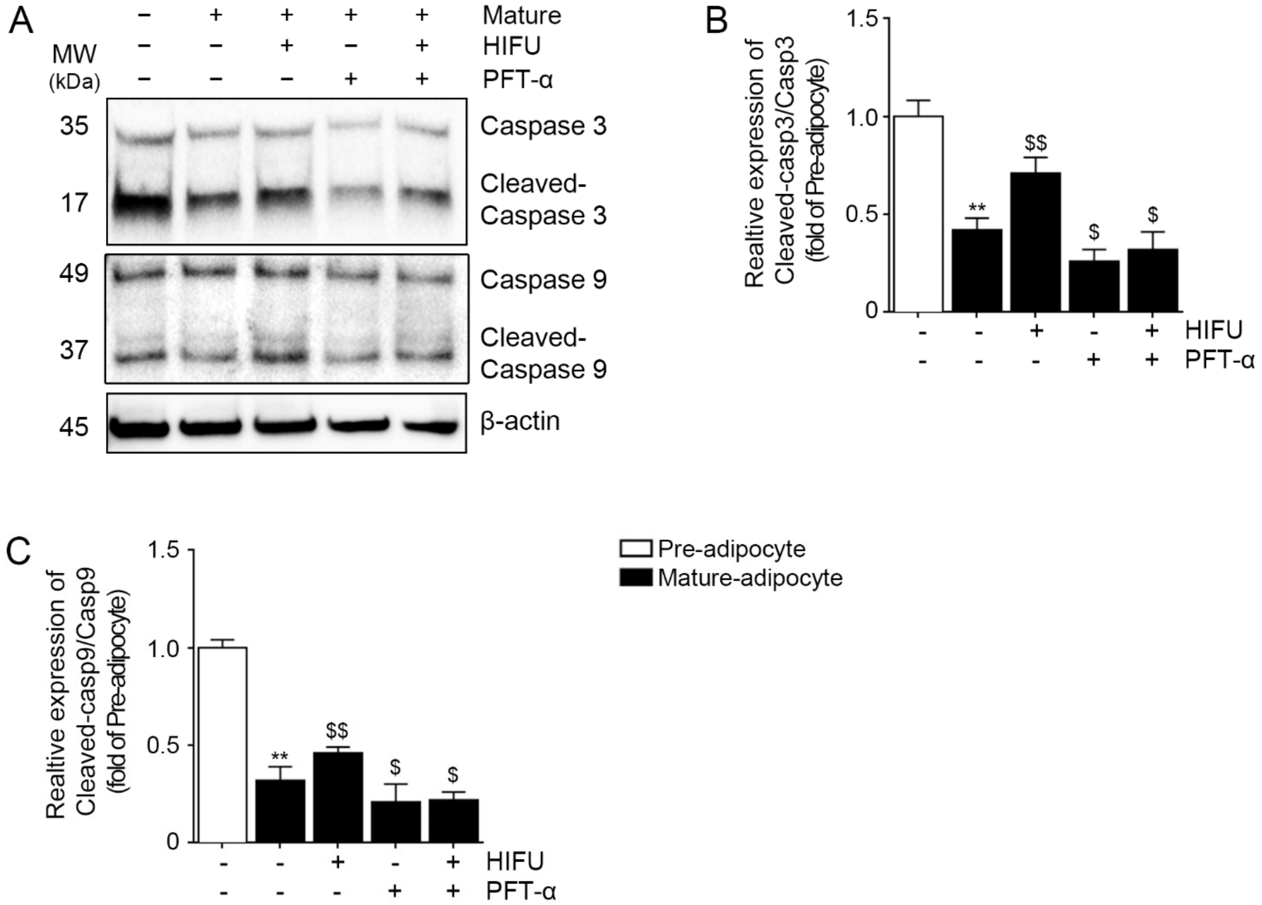

Figure S4. Regulation of apoptosis in adipocytes by HIFU treatment. (A) The protein expression levels of caspase 3 and caspase 9 were analyzed by western blot. (B and C) The quantitative graphs represent the results of the western blot analysis. The protein expression ratios of cleaved caspase 3/caspase 3 (B) and cleaved caspase 9/caspase 9 (C) were increased by HIFU treatment and decreased modulating p53. The data are presented as the mean ± standard deviation; **, $p < 0.01$ Mature-adipocyte *vs.* Pre-adipocyte; $, $p < 0.05$ or $$, $p < 0.01$ *vs.* Mature-adipocyte (Mann–Whitney U test). HIFU, high-intensity focused ultrasound; MW, molecular weight; PFT-α, pifithrin-α hydrobromide.

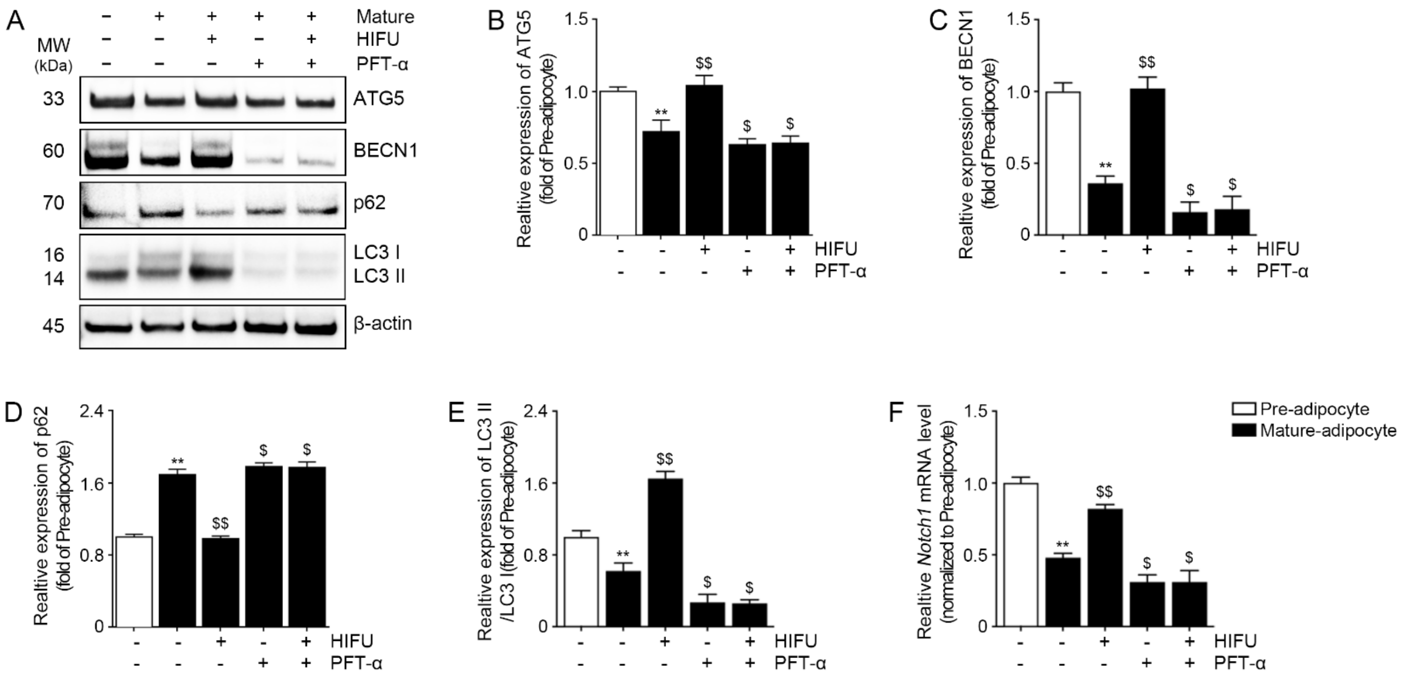

Figure S5. Regulation of autophagy in adipocytes by HIFU treatment. (A) The protein expression levels of autophagy-related markers were analyzed by western blot. (B–E) The quantitative graphs represent the results of the western blot analysis. The protein expression of ATG5 (B), BECN1 (C), and p62 (D) and the ratio of LC3II to LC3I (E) were regulated by HIFU treatment and p53. The data are presented as the mean ± standard deviation; **, $p < 0.01$ Mature-adipocyte *vs.* Pre-adipocyte; $, $p < 0.05$ or $$, $p < 0.01$ *vs.* Mature-adipocyte (Mann–Whitney U test). ATG5, autophagy related 5; BECN1, beclin-1; HFD, high-fat diet; HIFU, high-intensity focused ultrasound; LC3, microtubule-associated protein 1A/1B-light chain 3; MW, molecular weight; PFT-*α*, pifithrin-*α* hydrobromide.
